# Poisoning by Snails

**Published:** 1843-05

**Authors:** 


*Poisoning by Snails.*—A family of peasants living in the commune of Clermont, near Toulouse, fell a sacrifice to poisoning by snails. The physician who attended them communicated the details to the 'Journal de Toulouse:'—'From what I collected concerning the circumstances which preceded the disease, and those which accompanied it, and from the symptoms which I myself witnessed, I had no difficulty in recognizing a case of poisoning like those occasioned by narcotico-acrid vegetables, such as belladonna, hyoscyamus, thorn-apple, &c. No doubt remained in my mind as to the cause of this terrible disease, as soon as I knew that the snails eaten had been collected in the bushes called in French *redout*, but in the patois of the country, *roudout*. Every one knows that the leaves and young shoots are a poison to the domestic animals which browse on them, and that they kill them, after causing giddiness, and a kind of epileptic attack; but a fact which is not known is, that the flesh of these animals may occasion the greatest danger, and even death itself. Symptoms like those which I have just witnessed are rare; but it is common to see among our peasants indisposition caused by snails, which comes from their eating them as soon as they are gathered. The example of the ancient Romans should be followed, and these animals should not be brought to table until they have been kept six months or a year, feeding them with bran and wild thyme. This is the way also to make them fatter and more savory.—*New York Lancet*, from *Gazette Medicale.*

